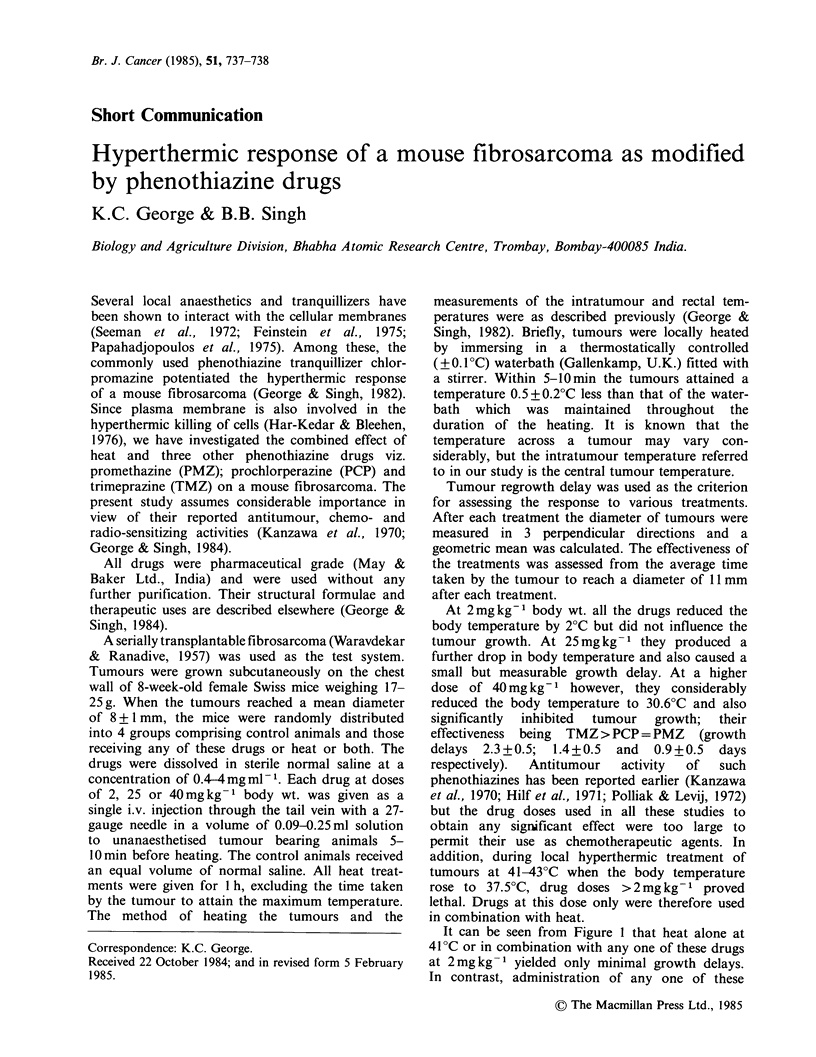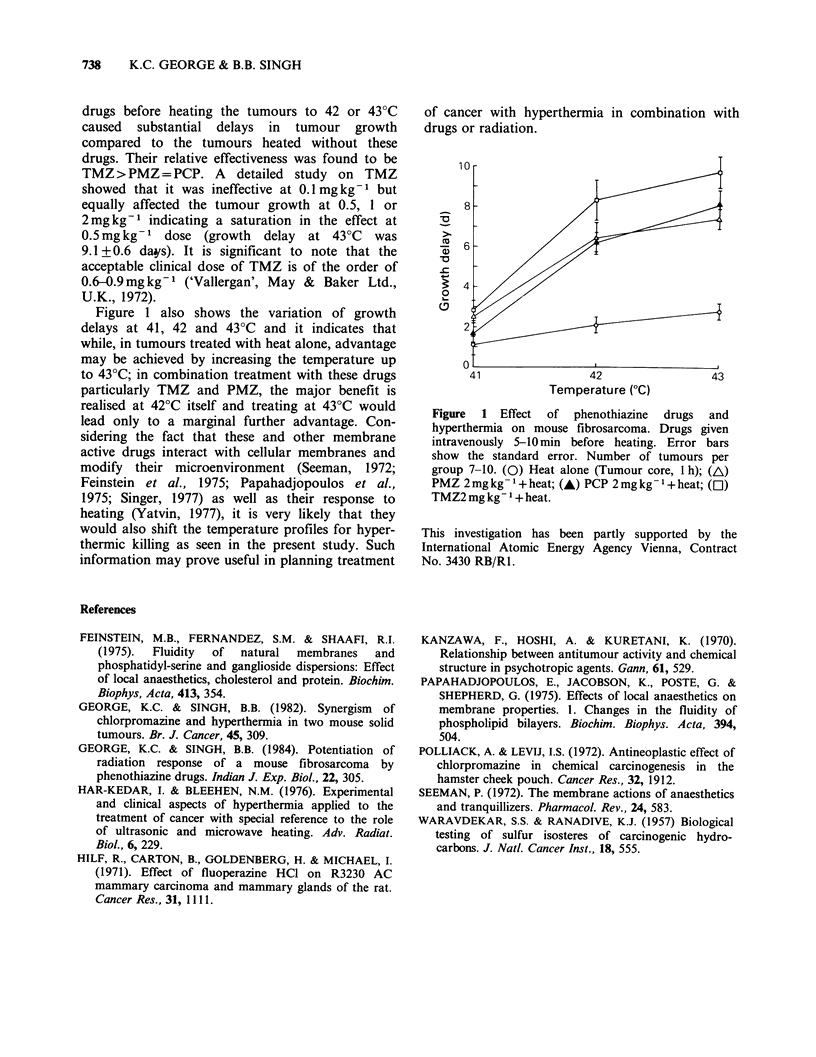# Hyperthermic response of a mouse fibrosarcoma as modified by phenothiazine drugs.

**DOI:** 10.1038/bjc.1985.111

**Published:** 1985-05

**Authors:** K. C. George, B. B. Singh


*Br. J. Cancer* (1985), **51**, 737–738

### Short Communication

### Hyperthermic response of a mouse fibrosarcoma as modified by phenothiazine drugs

K.C. George & B.B. Singh

*Biology and Agriculture Division, Bhabha Atomic Research Centre, Trombay, Bombay-400085 India.*

Several local anaesthetics and tranquillizers have been shown to interact with the cellular membranes (Seeman *et al.*, 1972; Feinstein *et al.*, 1975; Papahadjopoulos *et al.*, 1975). Among these, the commonly used phenothiazine tranquillizer chlorpromazine potentiated the hyperthermic response of a mouse fibrosarcoma (George & Singh, 1982). Since plasma membrane is also involved in the hyperthermic killing of cells (Har-Kedar & Bleehen, 1976), we have investigated the combined effect of heat and three other phenothiazine drugs viz. promethazine (PMZ); prochlorperazine (PCP) and trimeprazine (TMZ) on a mouse fibrosarcoma. The present study assumes considerable importance in view of their reported antitumour, chemo- and radio-sensitizing activities (Kanzawa *et al.*, 1970; George & Singh, 1984).

All drugs were pharmaceutical grade (May & Baker Ltd., India) and were used without any further purification. Their structural formulae and therapeutic uses are described elsewhere (George & Singh, 1984).

A serially transplantable fibrosarcoma (Waravdekar & Ranadive, 1957) was used as the test system. Tumours were grown subcutaneously on the chest wall of 8-week-old female Swiss mice weighing 17–25 g. When the tumours reached a mean diameter of $8 \pm 1$ mm, the mice were randomly distributed into 4 groups comprising control animals and those receiving any of these drugs or heat or both. The drugs were dissolved in sterile normal saline at a concentration of 0.4–4 mg ml$^{-1}$. Each drug at doses of 2, 25 or 40 mg kg$^{-1}$ body wt. was given as a single i.v. injection through the tail vein with a 27-gauge needle in a volume of 0.09–0.25 ml solution to unanaesthetised tumour bearing animals 5–10 min before heating. The control animals received an equal volume of normal saline. All heat treatments were given for 1 h, excluding the time taken by the tumour to attain the maximum temperature. The method of heating the tumours and the measurements of the intratumour and rectal temperatures were as described previously (George & Singh, 1982). Briefly, tumours were locally heated by immersing in a thermostatically controlled ($\pm 0.1$°C) waterbath (Gallenkamp, U.K.) fitted with a stirrer. Within 5–10 min the tumours attained a temperature $0.5 \pm 0.2$°C less than that of the waterbath which was maintained throughout the duration of the heating. It is known that the temperature across a tumour may vary considerably, but the intratumour temperature referred to in our study is the central tumour temperature.

Tumour regrowth delay was used as the criterion for assessing the response to various treatments. After each treatment the diameter of tumours were measured in 3 perpendicular directions and a geometric mean was calculated. The effectiveness of the treatments was assessed from the average time taken by the tumour to reach a diameter of 11 mm after each treatment.

At 2 mg kg$^{-1}$ body wt. all the drugs reduced the body temperature by 2°C but did not influence the tumour growth. At 25 mg kg$^{-1}$ they produced a further drop in body temperature and also caused a small but measurable growth delay. At a higher dose of 40 mg kg$^{-1}$ however, they considerably reduced the body temperature to 30.6°C and also significantly inhibited tumour growth; their effectiveness being TMZ > PCP = PMZ (growth delays $2.3 \pm 0.5$; $1.4 \pm 0.5$ and $0.9 \pm 0.5$ days respectively). Antitumour activity of such phenothiazines has been reported earlier (Kanzawa *et al.*, 1970; Hilf *et al.*, 1971; Polliak & Levij, 1972) but the drug doses used in all these studies to obtain any significant effect were too large to permit their use as chemotherapeutic agents. In addition, during local hyperthermic treatment of tumours at 41–43°C when the body temperature rose to 37.5°C, drug doses > 2 mg kg$^{-1}$ proved lethal. Drugs at this dose only were therefore used in combination with heat.

It can be seen from Figure 1 that heat alone at 41°C or in combination with any one of these drugs at 2 mg kg$^{-1}$ yielded only minimal growth delays. In contrast, administration of any one of these

Correspondence: K.C. George.
Received 22 October 1984; and in revised form 5 February 1985.

© The Macmillan Press Ltd., 1985

drugs before heating the tumours to 42 or 43°C caused substantial delays in tumour growth compared to the tumours heated without these drugs. Their relative effectiveness was found to be TMZ>PMZ=PCP. A detailed study on TMZ showed that it was ineffective at $0.1\,mg\,kg^{-1}$ but equally affected the tumour growth at 0.5, 1 or $2\,mg\,kg^{-1}$ indicating a saturation in the effect at $0.5\,mg\,kg^{-1}$ dose (growth delay at 43°C was $9.1 \pm 0.6$ days). It is significant to note that the acceptable clinical dose of TMZ is of the order of $0.6–0.9\,mg\,kg^{-1}$ ('Vallergan', May & Baker Ltd., U.K., 1972).

Figure 1 also shows the variation of growth delays at 41, 42 and 43°C and it indicates that while, in tumours treated with heat alone, advantage may be achieved by increasing the temperature up to 43°C; in combination treatment with these drugs particularly TMZ and PMZ, the major benefit is realised at 42°C itself and treating at 43°C would lead only to a marginal further advantage. Considering the fact that these and other membrane active drugs interact with cellular membranes and modify their microenvironment (Seeman, 1972; Feinstein *et al.*, 1975; Papahadjopoulos *et al.*, 1975; Singer, 1977) as well as their response to heating (Yatvin, 1977), it is very likely that they would also shift the temperature profiles for hyperthermic killing as seen in the present study. Such information may prove useful in planning treatment of cancer with hyperthermia in combination with drugs or radiation.

**Figure 1** Effect of phenothiazine drugs and hyperthermia on mouse fibrosarcoma. Drugs given intravenously 5–10 min before heating. Error bars show the standard error. Number of tumours per group 7–10. (○) Heat alone (Tumour core, 1 h); (△) PMZ $2\,mg\,kg^{-1}$ + heat; (▲) PCP $2\,mg\,kg^{-1}$ + heat; (□) TMZ2 $mg\,kg^{-1}$ + heat.

This investigation has been partly supported by the International Atomic Energy Agency Vienna, Contract No. 3430 RB/R1.

**References**

FEINSTEIN, M.B., FERNANDEZ, S.M. & SHAAFI, R.I. (1975). Fluidity of natural membranes and phosphatidyl-serine and ganglioside dispersions: Effect of local anaesthetics, cholesterol and protein. *Biochim. Biophys, Acta,* **413,** 354.

GEORGE, K.C. & SINGH, B.B. (1982). Synergism of chlorpromazine and hyperthermia in two mouse solid tumours. *Br. J. Cancer,* **45,** 309.

GEORGE, K.C. & SINGH, B.B. (1984). Potentiation of radiation response of a mouse fibrosarcoma by phenothiazine drugs. *Indian J. Exp. Biol.,* **22,** 305.

HAR-KEDAR, I. & BLEEHEN, N.M. (1976). Experimental and clinical aspects of hyperthermia applied to the treatment of cancer with special reference to the role of ultrasonic and microwave heating. *Adv. Radiat. Biol.,* **6,** 229.

HILF, R., CARTON, B., GOLDENBERG, H. & MICHAEL, I. (1971). Effect of fluoperazine HCl on R3230 AC mammary carcinoma and mammary glands of the rat. *Cancer Res.,* **31,** 1111.

KANZAWA, F., HOSHI, A. & KURETANI, K. (1970). Relationship between antitumour activity and chemical structure in psychotropic agents. *Gann,* **61,** 529.

PAPAHADJOPOULOS, E., JACOBSON, K., POSTE, G. & SHEPHERD, G. (1975). Effects of local anaesthetics on membrane properties. 1. Changes in the fluidity of phospholipid bilayers. *Biochim. Biophys. Acta,* **394,** 504.

POLLIACK, A. & LEVIJ, I.S. (1972). Antineoplastic effect of chlorpromazine in chemical carcinogenesis in the hamster cheek pouch. *Cancer Res.,* **32,** 1912.

SEEMAN, P. (1972). The membrane actions of anaesthetics and tranquillizers. *Pharmacol. Rev.,* **24,** 583.

WARAVDEKAR, S.S. & RANADIVE, K.J. (1957) Biological testing of sulfur isosteres of carcinogenic hydrocarbons. *J. Natl. Cancer Inst.,* **18,** 555.